# Impact of maternal energy drink consumption during gestation and lactation on brain health in neonatal Wistar albino rats

**DOI:** 10.1038/s41598-025-16338-1

**Published:** 2025-08-22

**Authors:** Marwa Moustafa Mohamed, Doaa S. R. Khafaga, Hamed A. Daboun, Heba Ali Abd El-Rahman, Mohamed A. El Desouky

**Affiliations:** 1 Chemistry Department, Biochemistry Division, Faculty of Science, Cairo University, Giza, 12613 Egypt; 2 Department of Basic Medical Sciences, Health Sector, Galala University, New Galala City, 43511 Suez Egypt; 3 Department of Organic Chemistry, Faculty of Science, Cairo University, Giza, 12613 Egypt; 4 Department of Zoology, Faculty of Science, Cairo University, Giza, 12613 Egypt

**Keywords:** Brain, Energy drink, Neonates, Dopamine, Acetylcholinesterase, Wistar albino rats, Biochemistry, Biotechnology

## Abstract

Energy drinks are rapidly gaining prominence in the global beverage industry, with projected sales reaching $60 billion within the next five years. These beverages often contain high levels of caffeine and the amino acid taurine, among other ingredients. The increasing consumption of energy drinks by children has sparked concerns regarding potential caffeine toxicity. In the present study, an energy drink was administrated at doses of 5 ml/Kg or 10 ml/Kg body weight. The comet assay demonstrated a significant elevation in DNA damage, evidenced by increased % DNA in tail and olive tail moment in the energy drink groups. Additionally, there were notable elevation in malondialdehyde levels as an oxidative stress marker, while reduction in superoxide dismutase activity and glutathione levels as antioxidant markers in energy drink groups. Furthermore, acetylcholinesterase activity and dopamine levels were significantly decrease in the energy drink groups compared to the control group. The high-dose groups exhibited a more pronounced effect than the low-dose groups, indicating a dose-dependent adverse effect.

## Introduction

Energy drinks (EDs) are liquids that are advertised to boost all things considered mental and physical performance. The worldwide energy drink industry has risen tremendously over the last several decades, and it is anticipated to increase to $86.01 billion by 2026 from $53.01 billion in 2018^1^. As mentioned by the National Center for Complementary and Integrative Health, individuals 18 to 34 years old are the highest consumers of energy drinks, with one in three adolescents consuming them on a regular basis^1^. Since its initial release in the United States in 1997, energy drinks have become increasingly popular owing to aggressive marketing strategies aimed at athletes and adolescents^2,3^. This is concerning because a new study reveals that some of the components in energy drinks are related to neurological, harmful cardiovascular, dental, gastrointestinal, metabolic, and renal consequences^1^. The constituents of most energy drinks are the same: sugar, caffeine, vitamins B2, B3, B6, and B12, as well as non-nutritive stimulants like taurine, L-carnitine, D-glucuronolactone, guarana extract, inositol, ginseng, and yerba mate^4–6^. The US Food and Drug Administration (FDA) does not regulate energy drinks because the majority of their ingredients are easily accessible to consumers^7^. Caffeine, recognized as an adenosine receptor antagonist, serves as a stimulant capable of activating neuronal control pathways within both the central and peripheral nervous systems. Exceeding the FDA’s recommended intake of caffeine, which is 400 mg per day, has been associated with a heightened risk of agitation, insomnia, anxiety, and gastrointestinal issues^8^. A chemical substance found in many animal tissues; taurine is categorized as an amino sulfonic acid. It is thought to have several positive effects, including improving cognitive function and controlling how skeletal muscles contract^7,9^. According to a recent study, taurine may have antioxidant properties that could help athletes prevent oxidative muscle injury during vigorous exercise^10^. Glucuronolactone, a naturally occurring compound produced in minimal amounts within the body, is incorporated into energy drinks to enhance cognitive performance; however, there has been limited research examining the efficacy of this dietary supplement^7^. Recent studies indicate that certain dietary additives found in energy drinks, such as glucuronolactone, gluconolactone, and taurine, may possess neurotoxic properties^11^. Energy drinks are often high in sugar content, and excessive consumption has been associated with adverse effects, including irregular heart rate, fluctuating blood pressure, and obesity^12–16^. This study intended to examine the overall neurochemical and histological changes triggered by different dosages of energy drinks in the entire brain of neonatal Wistar albino rats, providing a preliminary evaluation of their effects.

## Materials and methods

### Chemicals

An energy drink (Red Bull^®^) was purchased from a local market in Giza, Egypt. The acetylcholinesterase activity assay kit (ELISA) was acquired from Sigma-Aldrich Company, while the (ELISA) kit of dopamine was acquired from Abcam Company (Cambridge, UK). Oxidative stress marker and antioxidant markers reagent kits had been supplied from Biodiagnostic, a company based in Giza, Egypt.

### Experimental animals

Eighteen female and nine male Wistar albino rats (*Rattus norvegicus*) (160–180 g) were purchased from the Faculty of Veterinary Medicine, Cairo University (Giza, Egypt). At the laboratory animal unit of the Zoology Department, Faculty of Science, Cairo University (Giza, Egypt) all animals were housed one week in suitable (65 × 25 × 15 cm) polypropylene cages with free food and water access, following a 12-hour light/dark cycle, controlled temperature (20–25 ˚C) and humidity (40–50%) to allow acclimatization to the experimental setting. The animal protocol was executed in compliance with the established standards for the care of experimental animals. Furthermore, the findings of this study are presented in alignment with the ARRIVE guidelines, and the research received approval from Cairo University-Institutional Animal Care and Use Committee (CU-IACUC), with approval number (CU/I/F/73/22); all experiments were performed by relevant guidelines and regulations.

### Experimental design

All rats were prepared for mating by housing two females with one male in a cage overnight. Vaginal smears were obtained from the female rats and examined the next morning. By using a pipette, a tiny small amount of saline was pushed into the rat’s vaginal opening, and two drops of the vaginal fluid which contained cell suspension, were put on a slide and then stained with 0.1% methylene blue. Slides were examined under a microscope with a magnification of 100x after drying. The presence of sperm in the vaginal smear is an indication for zero-day gestation. On the fifth day of gestation, all animals were split up randomly into three groups, each consisting of six pregnant rats, which were assigned as follows: Control group (*n* = 6): Received an oral dose of distilled water via gavage. Low-dose group (*n* = 6): Received Red Bull^®^ at a dose 5 ml/kg body weight daily from the fifth day to twenty-one day of gestation, followed by continued administration for twenty-one day during lactation^15,17,18^ via oral gavage according to guidelines from the Organization for Economic Co-operation and Development (OECD, 2000). High-dose group (*n* = 6): Received Red Bull^®^ at a dose 10 ml/kg body weight daily from the fifth day to twenty-one day of gestation, followed by continued administration for twenty-one day during lactation^15,17,18^ via oral gavage. We used Red Bull^®^ which contains (per 100 ml) 400 mg of taurine, 32 mg of caffeine, 240 mg of gluconolactone, 20 mg inositol, 8 mg of niacin, 11.3 g of sucrose and glucose, 2.4 mg of pantothenic acid, vitamins B2/B6/B12, citric acid, flavorings, colors.

### Sample collection

At the end of the experiment, the animals were euthanized by sudden decapitation, and then the brain tissue was removed from the skull. Part of each brain tissue was stored at −80 ˚C immediately for a comet assay to assess the damage to the intact DNA, determine the oxidative stress marker and antioxidant markers, acetylcholinesterase activity and dopamine levels, while the other part was stored in 10% formalin for histopathological examination.

### Biochemical analysis

#### Determination of oxidative stress marker and antioxidant markers

Oxidative stress marker as malondialdehyde (MDA) levels^19^and antioxidant markers as superoxide dismutase (SOD) activity^20^and reduced glutathione (GSH) levels^21^ in the brain tissue of neonates were determined using colorimetric method kits (Catalog Number: MD2529), (Catalog Number: SD2521), and (Catalog number: GR2511), respectively, according to the manufacturer^’^s guidelines and instructions.

#### Determination of acetylcholinesterase and dopamine in brain tissue

Brain tissues of neonates were used for the determination of acetylcholinesterase (AChE) activity using a colorimetric (ELISA) kit from Sigma-Aldrich Company (Catalog Number: MAK119) and dopamine (DA) levels using a colorimetric (ELISA) kit from Abcam Company (Catalogue Number: ab285238), according to the manufacturer’s guidelines and instructions.

#### Comet assay

Single-cell gel electrophoresis (SCGE), a comet assay, is widely used to identify DNA damage in certain cell types. Under a fluorescence microscope, damaged cellular DNA separates from uninjured DNA in an electrophoretic field to form a structure that resembles the tail of a comet. First, 100 ml of agarose (low melting point, 1%) in PBS at 37 ˚C was applied to the slides to create frosted slides. The agarose was then frozen, the tissues were centrifuged for five minutes at 2000 rpm, and the slides were again submerged in ice-cold PBS (step 1). Additionally, 100 ml of low-melting-point agarose was mixed with roughly 10 ml of this prepared tissue suspension, which was quickly spread on the frosted slide that had previously been coated with low-melting-point agarose (step 2). These produced agarose slides were then treated in cold lysis buffer at 4 ˚C for two hours, which caused the protein-depleted nucleus carrying supercoiled DNA to separate (step 3). To break the hydrogen bonds between the DNA strands and convert alkali-labile lesions into DNA strand breaks, the control and treated samples were next submerged in a strong alkaline solution (step 4). The slides were then put on a horizontal gel electrophoresis for 20 min using an electrophoresis buffer (pH 13.0, 25 V, and 300 mA) to create single-cell comets (step 5). To assure successful staining (step 6), these slides were then submerged three times in neutralization buffer (pH 7.5) to remove any remaining alkali and detergents. Last but not least, the slides were stained with an ethidium bromide stain (20 µg/ml) and then examined using a fluorescence microscope (step 7). Comets were identified as a diffused “cloud” of DNA material fluorescing around the nuclei or making a tail in the direction of the electric field. Tail lengths of 50 comets, in each slide, were measured from the edge of each nucleus using a calibrated ocular micrometer. This allowed for the capture of 100 images of the shapes of random comets for each slide were examined at 400x magnification, which were then analyzed by a computerized image analysis system that used TriTek Comet ScoreTM software (TriTek Corp., Sumerduck, VA, USA) to estimate the comet parameters (step 8)^15^. A computerized image analysis system was used to acquire images. Tail length was measured from the center of the head to the center of the tail, and calculations were performed using Eqs. (1), (2) and (3).

1 $$\% \text{DNA in tail}\:=\: 100 \times \text{Tail DNA Intensity}/\text{Cell DNA Intensity}$$

2 $$\text{Tail Moment}\:=\:\text{Tail length} \times \% \text{DNA in tail}$$

3 $$\text{Olive Tail Moment (OTM)} \:=\: \% \text{DNA in tail} \times \text{Tail Moment Length}$$

### Histopathological examination

Small slices of brain tissue were promptly collected for fixation in 10% formalin for 24 h. Subsequently, the samples underwent a series of processing procedures, which included washing with tap water, dehydration through increasing concentrations of ethanol, clearing with xylene, and embedding in paraffin wax with a melting point ranging from 56 to 60 °C. Tissue sections were then cut to a thickness of 5 μm and stained using hematoxylin and eosin. The paraffin sections were treated with Harris hematoxylin for 5 min, rinsed in running water to enhance the blue hue, and subsequently immersed in a 1% aqueous eosin solution for 2 min. After this staining process, the sections were rinsed, dried, cleared, and mounted in canada balsam^22–24^.

## Statistical analysis

The mean (µ) and standard deviation (SD) of the tests across all studied groups (*n* = 6) were calculated using SPSS Statistics version 25 and presented as µ ± SD. Differences between the groups were analyzed using ANOVA, followed by post-hoc LSD tests, with statistical significance set at *P* < 0.05.

## Results

### Oxidative stress marker and antioxidant markers

**Fig. 1:**
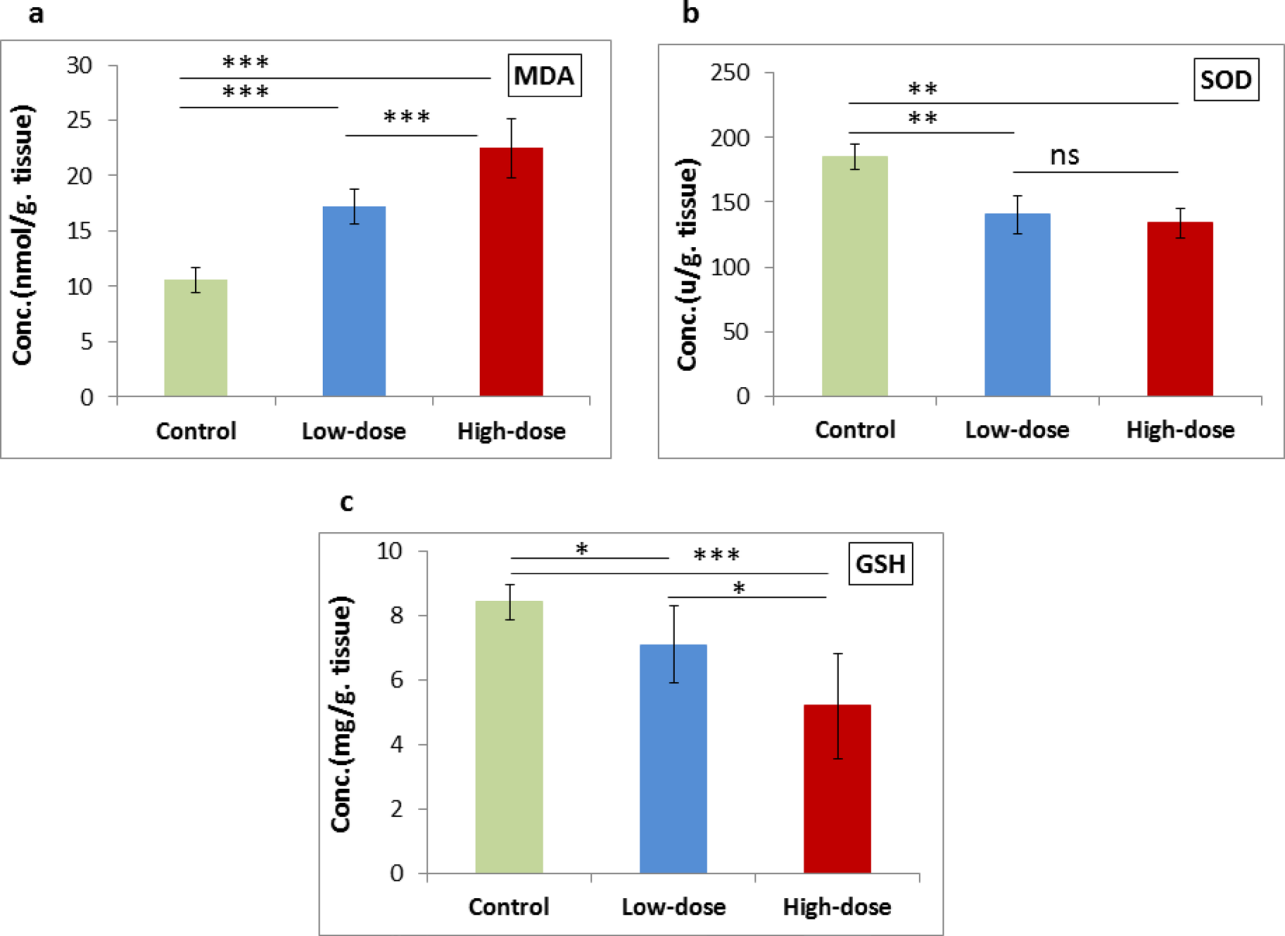
Effect of low- and high-dose ED consumption on **(a)** MDA, **(b)** SOD and **(c)** GSH in the brain of neonates; collected from control group, low-dose group (5 ml/kg) and high-dose group (10 ml/kg). Data are shown as Mean ± SD (*n* = 6). ***P < 0.001, **P < 0.01, *P < 0.05 and ns (non-significant).

MDA levels showed a highly significant increase (*P* < 0.001) when comparing the high-dose group with either the low-dose or control group, as well as when comparing the low-dose group with the control group, as illustrated in Fig. 1**(a)**. SOD activity showed a significant decrease (*P* = 0.001) in both the low-dose and high-dose groups compared to the control group. However, the decrease in SOD activity between the high-dose group and low-dose groups was non-significant (*P* = 0.414), as illustrated in Fig. 1**(b)**. A significant decrease in GSH levels was observed when comparing the low-dose with the control group (*P* = 0.032) and with the high-dose group (*P* = 0.046). Furthermore, a highly significant decrease (*P* < 0.001) was detected when comparing the high-dose group with the control group, as illustrated in Fig. 1**(c)**.

### Acetylcholinesterase and dopamine

**Fig. 2:**
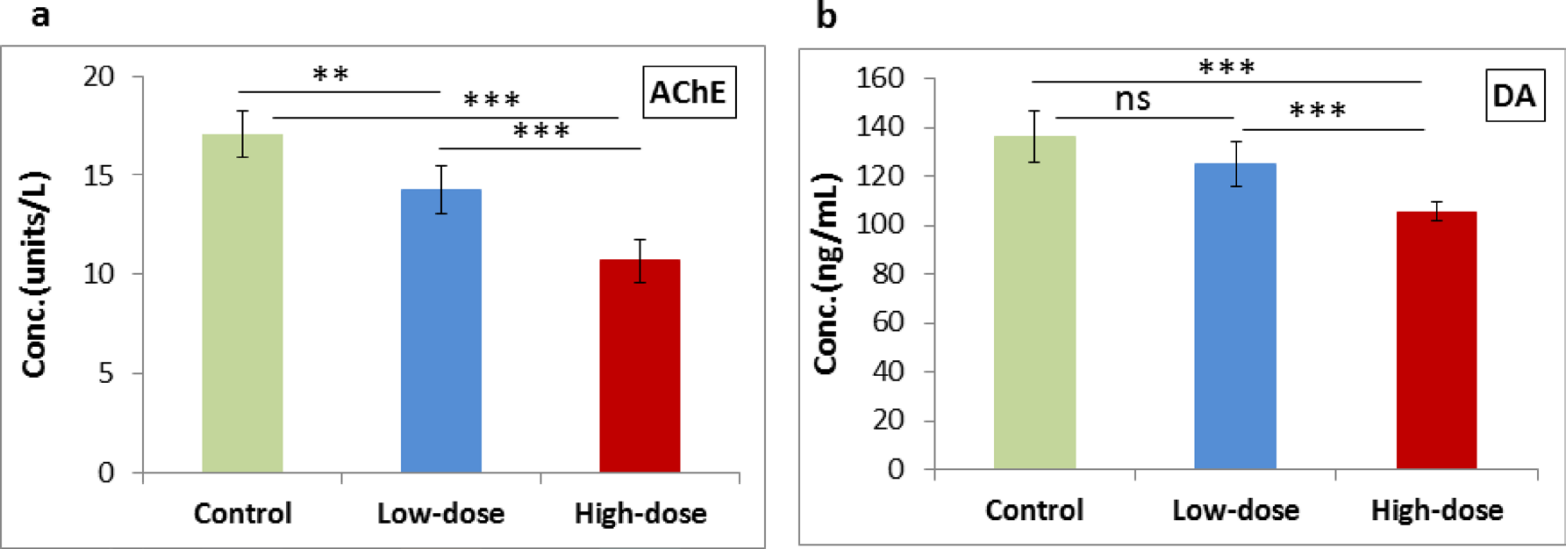
Effect of low- and high-dose ED consumption on **(a)** AChE and **(b)** DA in the brain of neonates; collected from control group, low-dose group (5 ml/kg) and high-dose group (10 ml/kg). Data are shown as Mean ± SD (*n* = 6). ***P < 0.001 , **P < 0.01 and ns (non-significant).

AChE activity and DA levels showed a highly significant decrease (*P* < 0.001) when comparing the high-dose group with either the control group or the low-dose group. Comparison between the low-dose group and control group revealed a significant decrease in AChE activity (*P* = 0.0025), while the decrease in DA levels was non-significant (*P* = 0.074). Furthermore, the high-dose group exhibited a significantly greater reduction in both AChE activity and DA levels compared to the low-dose, indicating a dose-dependent effect, as illustrated in Fig. 2.

### Comet assay

**Fig. 3:**
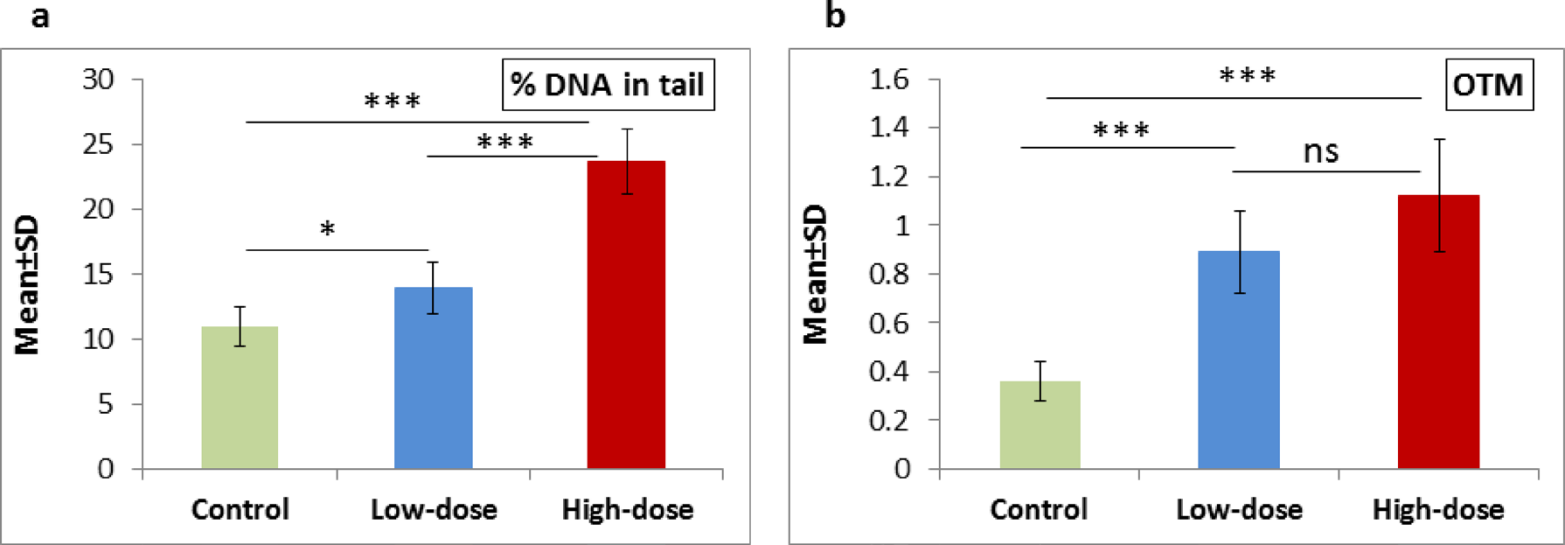
Effect of low- and high-dose ED consumption on **(a)** % DNA in tail and **(b)** OTM in the brain of neonates; collected from control group, low-dose group (5 ml/kg) and high-dose group (10 ml/kg). Data are shown as Mean ± SD (*n* = 6). ***P < 0.001, *P < 0.05 and ns (non-significant).

**Fig. 4:**
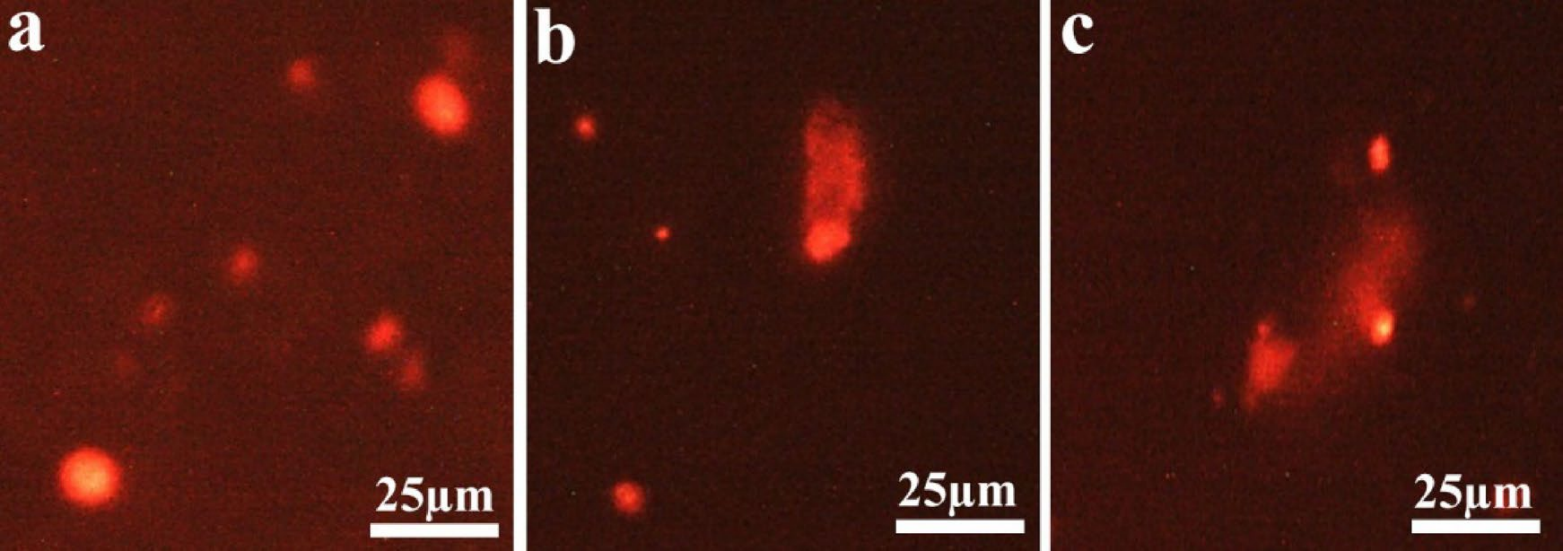
Fluorescence microscopy images for the effect of ED on the nucleus. **(a)**: Control group: Intact nucleus with no discernible DNA damage, **(b)**: Low-dose group (5 ml/kg) and **(c)**: High-dose group (10 ml/kg); Nucleus displaying DNA damage indicated by a comet tail.

A highly significant increase in % DNA in tail (*P* < 0.001) was observed when comparing the high-dose group with either the low-dose group or control group. Additionally, a significant increase (*P* = 0.013) was noted when comparing the low-dose group with the control group, as illustrated in Fig. 3**(a)**. In OTM, both the high-dose and low-dose groups showed a highly significant increase (*P* < 0.001) compared to the control group, while the increase between the high-dose group and low-dose group was a non-significant (*P* = 0.08), as illustrated in Fig. 3**(b)**. Figure 4 illustrates the DNA damage induced by ED exposure among the different study groups using fluorescence microscope.

### Histopathological examination

**Fig. 5:**
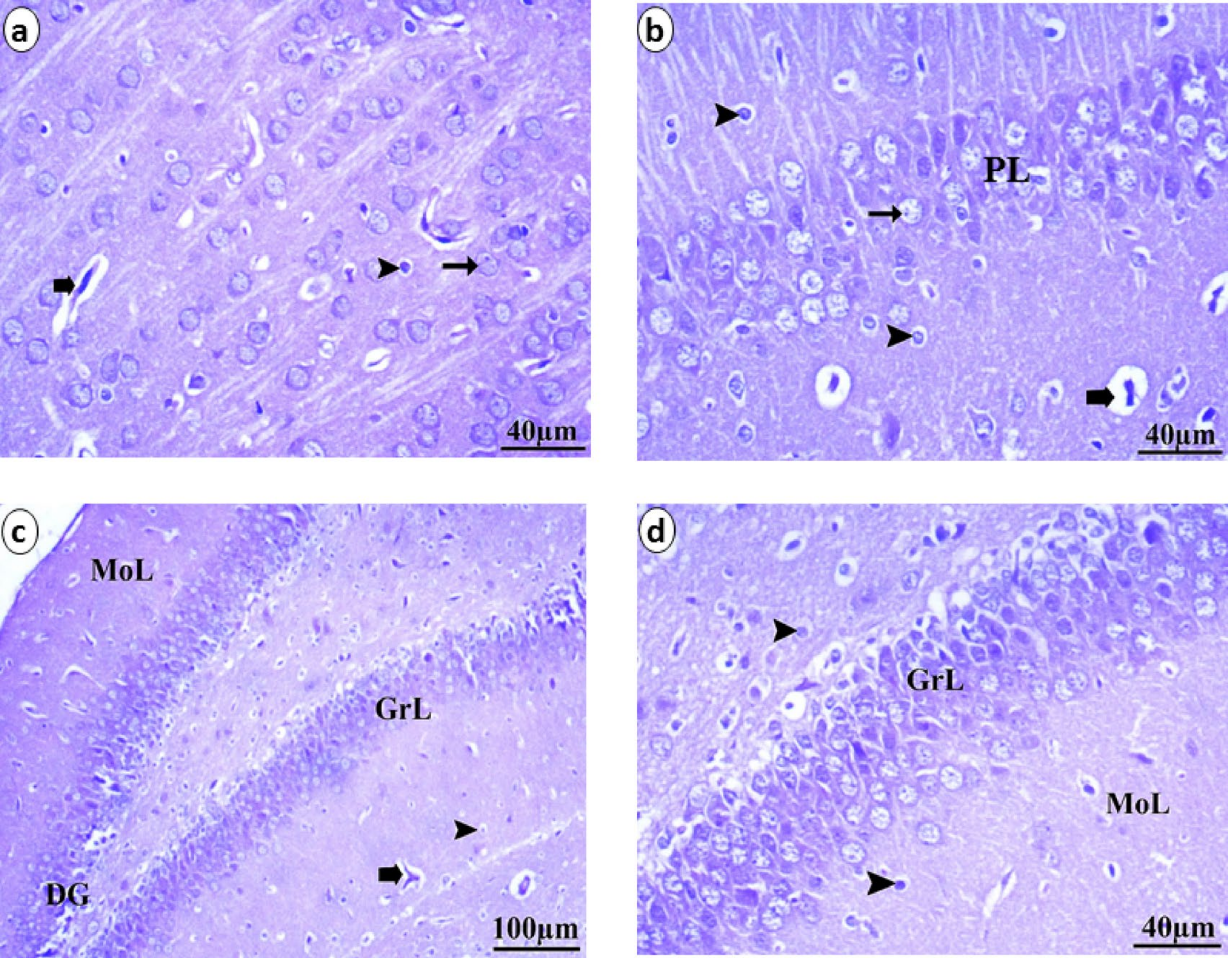
Histological analysis of neonatal brain tissue stained with H&E shows that the control group exhibits a normal structure in the cerebral cortex **(a&b)**. This includes healthy pyramidal cells (arow), microglia cells (arrowhead), and blood vessels (bold arrow). Additionally, the hippocampus **(c&d)** displays well-formed pyramidal cell layers (PL) and molecular layers (MoL). The dentate gyrus (DG) also presents a clearly defined granule cell layer (GrL) with appropriately aggregated granule cell bodies.

**Fig. 6:**
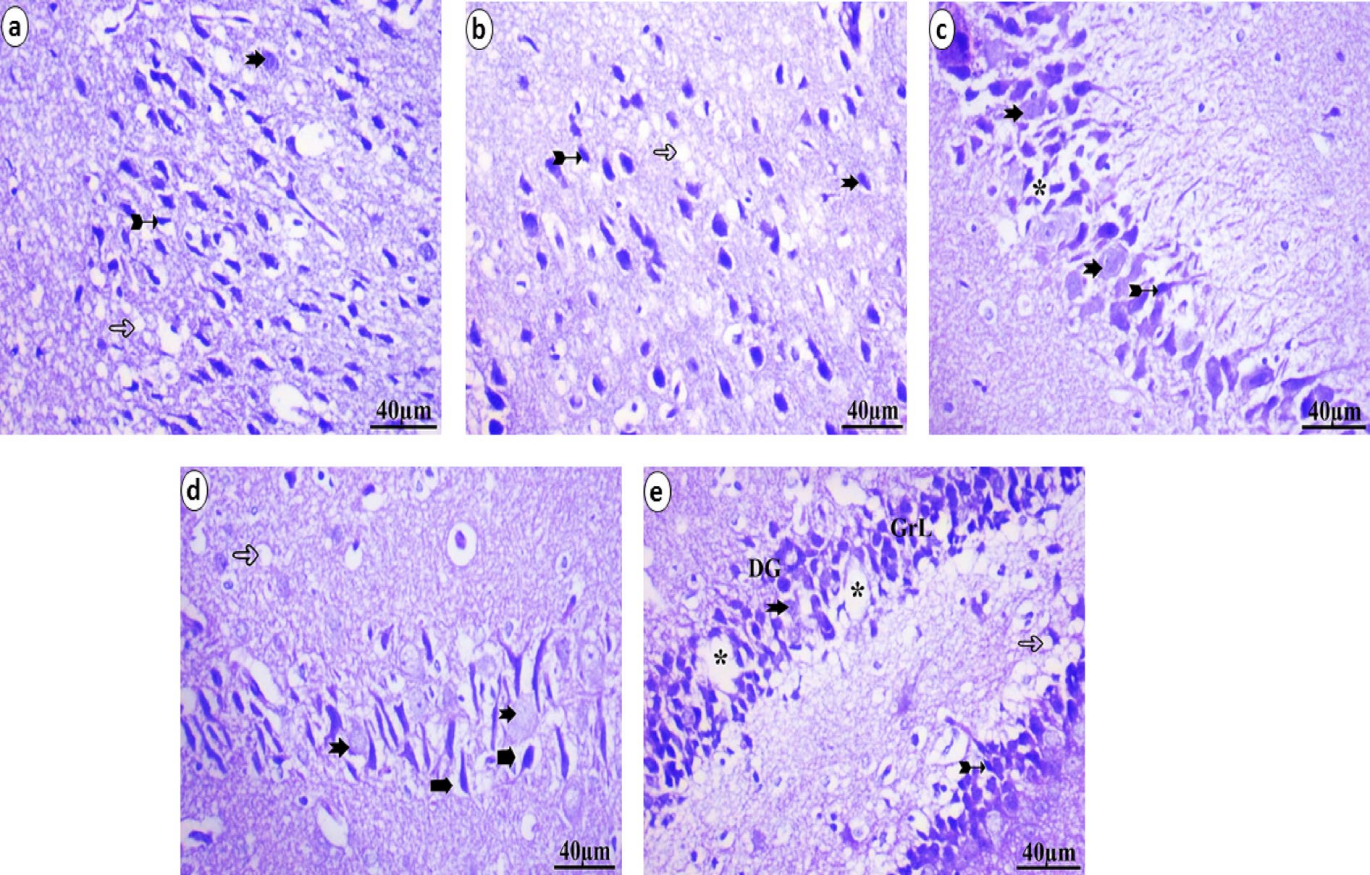
Histopathological examination of neonatal brain tissue following maternal ED intake at a low-dose (5 ml/kg) revealed several abnormalities. The cerebral cortex **(a&b)** and hipopocampus **(c&d)** showed vacuolation (hollow arrow), darkly stained pyknotic nuclei (bifid arrow), and ghost degenerated neurons (notch arrow). Additionally, the granule cell layer (GrL) of the dentate gyrus (DG) appeared disorganized with noticeable empty areas (*) **(e)**.

**Fig. 7:**
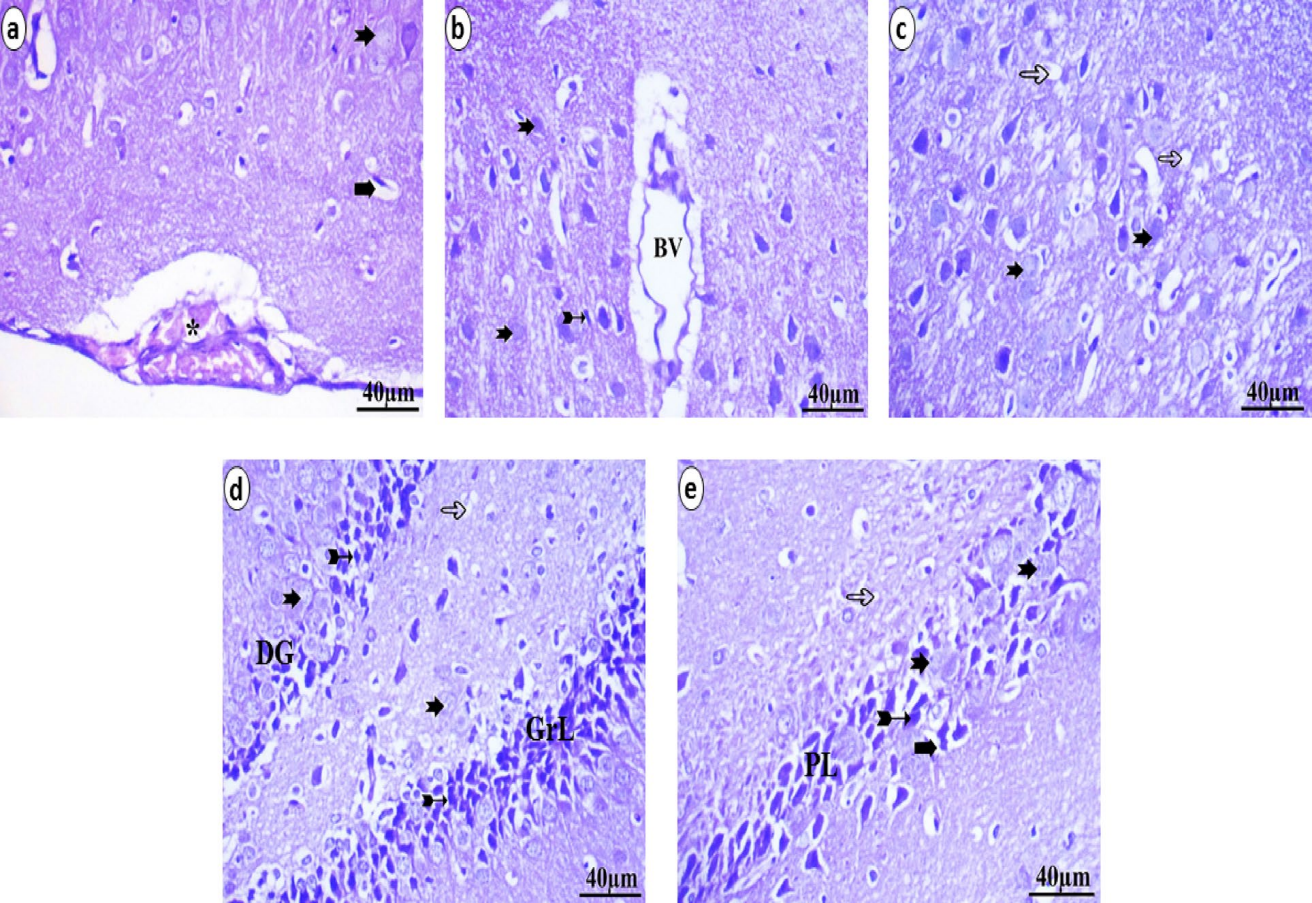
Histopathological examination of neonatal brain tissue after maternal ED intake at a high-dose (10 ml/kg) revealed numerous changes. These included neuropil vacuolation (hollow arrow), blood vessel dilation (BV), and pia mater congestion (*) within the layers of the prefrontal cortex **(a&b)**. The tissue also showed pyknotic nuclei (bifid arrow) surrounded by pericellular haloes (bold arrow) and ghost degenerated neurons (notch arrow). In the hippocampus **(c&d)**, both the pyramidal layer (PL) and the granular cell layer (GrL) of the dentate gyrus (DG) exhibited a disorganized appearance **(e)**.

The different areas of the neonate brain that were analyzed through histopathological methods were illustrated in Figs. 5, 6 and 7. The control group presented a normal histological pattern of the brain; a typical monomorphic pattern of neurons with long dendrites, microglia cells, and a normal form of pyramidal cells was detected. The ground material between nerve cells is normally occupied by uniform neuropil. The pyramidal cell layer and molecular layer of the hippocampus appear to be in good shape. Pyramidal neurons in the PL have small cell bodies with vesicular nuclei and scant cytoplasm and are closely packed and structured. In the molecular layer, glial cells are present. The granule cell layer in the dentate gyrus is well defined. The granular layer displays the aggregation of granule cell bodies, which range from spherical to oval, as illustrated in Fig. 5. The low-dose group (5 ml/kg) presented vacuolation, decreased numbers of neurons, and darkly stained pyknotic nuclei. Presence of ghost degenerated neurons. Pyramidal neuron cell bodies are disordered and loosely packed in the hippocampus; they appear black, shrunken and contain pyknotic nuclei with pericellular haloes. The granular cell layer of the dentate gyrus was similarly disordered with empty areas, as illustrated in Fig. 6. The high-dose group (10 ml/kg) displays a variety of histopathological changes, the most notable of which are a high degree of neuropil vacuolation. There was a degree of blood vessel dilation and pia mater congestion in the layers of the prefrontal cortex. The dark, shrunken neuronal cell bodies and highly pigmented pyknotic nuclei may be observed within the pericellular haloes and ghost degenerated neurons. In the hippocampus, the neurons of the pyramidal layer are disorganized and loosely packed; they have a dark, shrunken appearance and contain pyknotic nuclei and neurons surrounded by pericellular haloes and degenerated neurons. The granular cell layer from the dentate gyrus had the same disorganized appearance. Vacuolation of the neuropil was seen, as illustrated in Fig. 7.

## Discussion

The Food and Drug Administration characterizes EDs as a category of liquid products that generally contain caffeine, with or without additional ingredients. Caffeine, additional sugars, other chemicals, and legal stimulants like taurine, L-carnitine, and guarana are frequently found in these drinks. These legal stimulants can increase energy, alertness, and focus while also raising heart rate, blood pressure, and breathing. EDs are promoted as products that improve mental acuity and physical performance. Red Bull, Monster, NOS, Rockstar, Lucozade, Eastroc Super Drink, and Bang Energy are a few well-known brands of energy beverages. These drinks are very popular among young people since they can rapidly increase energy levels, increase attentiveness, and enhance performance in sports or academics^25^.

Because of its strong capacity to permeate membranes, including the placental and blood-brain barriers, it spreads quickly to all bodily tissues. A high milk-to-serum concentration ratio is the result of caffeine’s passive diffusion across the placenta, its widespread distribution in fetal tissues, and its easy excretion in breast milk^26^.

Through increased catecholamine production, caffeine can promote vasoconstriction in the fetus, which lowers placental and uterine blood flow and results in fetal hypoxia^27^. Caffeine use during pregnancy altered the overall infant development trajectory from birth to age eight in a nationwide cohort of pregnant women by raising the risk of excessive infant growth and childhood overweight^26^. Caffeine’s effects are dependent on its metabolism rates, which might vary depending on a person’s weight, gender, genetic background, and the pace at which CP450 enzymes are induced in the liver. Excessive caffeine use can cause several negative side effects, including headache, nausea, tachycardia, irritability, sleeplessness, tremors in the extremities, and even an anxiety attack^28^. The fetus does not express the primary enzymes that deactivate caffeine, and it has been observed that caffeine metabolites accumulate in the fetal brain^29^. Brain tissue is the most complex organ, regulating cognitive processes, sensory perception, motor function, memory, communication, and environmental reactions. Proper nutrition is crucial for optimal development, especially during gestation and lactation stages. Adequate maternal nutrition during pregnancy and breastfeeding significantly impacts the long-term health of both mother and baby, preventing disorders in brain tissue^30^.

The central nervous system (CNS) was a critical focus of our research due to its high concentration of polyunsaturated fatty acids, making it particularly susceptible to damage from oxidative stress. Perinatal exposure to ED resulted in oxidative stress within the cerebral cortex, cerebellum, and medulla oblongata of newborns at 21- and 35-day post-birth. Notably, all three brain regions exhibited a marked rise in the levels of lipid peroxidation, alongside a reduction in antioxidant defenses, specifically GSH and SOD, in the offspring of mice subjected to both low- and high-doses of the ED. Furthermore, recent studies have indicated that the intake of EDs can also provoke oxidative stress in various brain regions of adult rats^16^. In the present study, MDA levels highly significant increased in both the ED groups compared with the control group, with the high-dose group exhibiting a significantly higher MDA level than the low-dose group. This implies greater lipid peroxidation and oxidative stress at higher dose, while there was a decrease of SOD and GSH antioxidant markers of the neonates’ brain tissues in the ED groups compared with the control group. GSH levels showed a significant further decreased in the high-dose group compared with the low-dose group, indicating diminished antioxidant defense with increased ED exposure, which agree with Hanna et al.^31^ study, which indicated that MDA levels increase while SOD activity and GSH levels decline in brain tissues of the pregnant rats. The influence of EDs on the CNS of neonatal mice cannot be ascribed to a singular component. This perspective is corroborated by the research conducted by Valle et al.^18^which indicated that an oxidative imbalance was observed in both the prefrontal cortex and the hippocampus of adult rats subjected to a mixture of caffeine and taurine. Valle et al.^18^ attributed their observations to a reduction in antioxidant levels and an increase in free radicals, aligning with our own results. Similarly, human SH-SY5Y cells exposed to guarana, either alone or in conjunction with caffeine (0.125–2 mg/ml) or taurine (1–16 mg/ml), exhibited diminished antioxidant defenses^32^. Furthermore, subchronic caffeine exposure led to a decrease in SOD in the striatum and glutathione peroxidase (GPx) activity in the prefrontal cortex, hippocampus, and striatum of adult rats^18^. Conversely, some studies have suggested that caffeine may play a protective role against oxidative stress. Caffeine is capable of mitigating oxidative stress through its function as a hydroxyl radical receptor^33^. Noschang et al.^34^ demonstrated that chronic caffeine intake could reduce lipid peroxidation and enhance antioxidant levels in the brains of rats. Additionally, taurine has exhibited radical scavenging properties in vitro^35^ and antioxidant effects in vivo^36^. In Valle et al.‘s study^18^the administration of taurine over a 28-days resulted in decreased antioxidant enzyme activities in the brains of adult rats. Moreover, rats that received a combination of taurine and caffeine experienced oxidative stress and a reduction in antioxidant defenses across various brain regions^18^. It can be inferred that the interaction of caffeine and taurine may lead to oxidative stress within the brain, which could account for the observed increase in lipid peroxidation and the decrease in antioxidants in the brains of neonates in our research.

Inhibiting AChE is currently a common pharmacological strategy for several conditions, such as myasthenia gravis, postoperative use, Alzheimer’s disease, and certain eye diseases^37^. AChE plays an important role in the formation of the apoptosome and the regulation of the apoptotic process. Decrease activity of AChE produces substantial DNA damage, reactive oxygen stress (ROS), and an increase in micronuclei frequencies, leading to the increased risk of genotoxic processes^37^. Our findings concerning acetylcholine align closely with those reported by Abdelwahab et al.^38^who observed a notable reduction in acetylcholinesterase enzymatic activity, leading to an accumulation of acetylcholine at both muscarinic and nicotinic receptors. Similarly, Ebuehi et al.^39^ reported a significant increase in acetylcholine levels in the brain tissue of rats after administering 5 ml of Red Bull or Power Hoarse over 35 days. DA belongs to the catecholamine family, which plays a pivotal role as a neurotransmitter in the central nervous, hormonal, renal, and cardiovascular systems. The DA disorders, such as Parkinson’s and Alzheimer’s, may be brought on by bodily inadequacies. Inadequate DA levels are linked to these conditions as well as Tourette’s syndrome, schizophrenia, and hyperactivity disorder^40^. The consumption of EDs has been shown to significantly lower the levels of dopamine in various regions of the brain. This phenomenon can be attributed to the effects of caffeine, which acts as an inhibitor of adenosine receptors in the brain^31^. Such inhibition results in an increased influx of calcium ions into neuronal cells, leading to the release of neurotransmitters that are stored in vesicles within presynaptic cells, facilitated by the protein synapsin 1. Consequently, there is a reduction in the overall quantity of neurotransmitters present within these cells^31^. These findings are consistent with earlier research indicating a notable decline in brain dopamine levels following the intake of energy drinks^31^. Nevertheless, there exists contradictory evidence concerning the influence of coffee on the enhancement of dopamine levels in brain tissue^41^. Both markers were significantly lower in the ED groups compared with the control group. The high-dose group showed more substantial reductions than the low-dose group, highlighting greater neurochemical disruption at higher intake levels, indicating a dose-dependent neurochemical effect.

In recent decades, single-cell gel electrophoresis, commonly referred to as the comet assay, has become a commonly used method for assessing DNA damage^42^. Its applications span various fields, including genotoxicity testing, molecular epidemiology, human biomonitoring, ecogenotoxicology, and essential research focused on DNA damage and repair mechanisms^43^. The most effective method for characterizing DNA break frequencies is proposed to be represented as a % DNA in tail, as this allows for a clearer observation of the damage caused by the aforementioned comet. Nevertheless, Møller et al.^44^ indicate that numerous researchers continue to prefer the use of tail moments. In reality, the conditions of the assay exert a comparable influence on both descriptors^45,46^. In the present study, the % DNA in tail and OTM were higher in the ED groups than in the control group, the high-dose group exhibiting more DNA damage. The % DNA in tail was significantly higher in the high-dose group than in the low-dose group. In our present study, the high-dose group demonstrated more severe histopathological alterations compared with the low-dose group, indicating more profound neurodegenerative effects. Consistent with our findings, Abdelwahab et al.^38^ documented that exposure to EDs resulted in histological changes in brain tissue, characterized by numerous apoptotic cells with small, darkly stained nuclei and surrounding empty spaces, as well as congested and dilated blood vessels. Additionally, Sayed^47^ noted that rats treated with Red Bull exhibited neurodegenerative alterations in the cerebral cortex, including intracytoplasmic vacuoles in neuronal cells, shrunken nuclei with dark staining, and changes in chromatin lysis. Furthermore, our results corroborate those of Salih et al.^48^. Energy drinks, characterized by elevated caffeine levels, both doses of EDs negatively affect may cause DNA damage in neonatal brain tissue, but the high dose induces significantly more oxidative stress. Caffeine enhances metabolic activity, resulting in the generation of reactive oxygen species that exceed the brain’s antioxidant defenses, especially when the activities of antioxidant enzymes are diminished. Oxidative stress causes damage to cellular components, including DNA, as evidenced by an increase in DNA strand breaks identified through the comet assay. The consequences may result in lasting cognitive and behavioral impairments. The dose-dependent findings emphasize the amplified risk associated with higher levels of ED consumption during pregnancy and lactation.

## Conclusion

This study showed that consumption of maternal energy drinks during gestation and lactation may harm newborns’ neurodevelopment. The comet assay showed that brain tissues have more DNA damage in the ED groups, which supports this hypothesis. Increased oxidative stress in the brain of the neonates is indicated by high malondialdehyde levels while antioxidant markers decrease. The decrease of acetylcholinesterase activity and dopamine levels in energy drink groups suggest possible neurochemical imbalances that may impact cognition and behavior. The serious public health risks of energy drink usage by pregnant people necessitate further investigation into the long-term consequences of these compounds on fetal and neonatal health. The need to reevaluate energy drink intake guidelines for those at risk is also emphasized. The current study employed a whole-brain analysis for neurochemicals, which fails to consider region-specific effects. Future research will focus on region-specific analyses to enhance comprehension.

## Data Availability

The data presented in this study are available on request from the corresponding author.
